# Effectivity of Two Cell Proliferation Markers in Brain of a Songbird Zebra Finch

**DOI:** 10.3390/biology9110356

**Published:** 2020-10-25

**Authors:** Lubica Kubikova, Justina Polomova, Viktoria Mikulaskova, Kristina Lukacova

**Affiliations:** 1 Institute of Animal Biochemistry and Genetics, Centre of Biosciences, Slovak Academy of Sciences, Dubravska Cesta 9, 840 05 Bratislava, Slovakia; Justina.Polomova@savba.sk (J.P.); Viktoria.Mikulaskova@gmail.com (V.M.); Kristina.Lukacova@savba.sk (K.L.); 2 Institute of Biochemistry and Microbiology, Faculty of Chemical and Food Technology, Slovak University of Technology in Bratislava, Radlinského 9, 812 37 Bratislava, Slovakia

**Keywords:** BrdU, EdU, ventricular zone, neurogenesis, click chemistry, liver, intestine, base analog

## Abstract

**Simple Summary:**

The present study compared the effectivity of two cell proliferation markers, BrdU and EdU, in the brain neurogenic zone of the songbird zebra finch. It shows their saturation doses, that BrdU labels more cells than the equimolar dose of EdU, and that both markers can be reliably detected in the same brain.

**Abstract:**

There are two most heavily used markers of cell proliferation, thymidine analogues 5-bromo-2′-deoxyuridine (BrdU) and 5-ethynyl-2′-deoxyuridine (EdU) that are incorporated into the DNA during its synthesis. In neurosciences, they are often used consecutively in the same animal to detect neuronal populations arising at multiple time points, their migration and incorporation. The effectivity of these markers, however, is not well established. Here, we studied the effectivity of equimolar doses of BrdU and EdU to label new cells and looked for the dose that will label the highest number of proliferating cells in the neurogenic ventricular zone (VZ) of adult songbirds. We found that, in male zebra finches (*Taeniopygia guttata*), the equimolar doses of BrdU and EdU did not label the same number of cells, with BrdU being more effective than EdU. Similarly, in liver, BrdU was more effective. The saturation of the detected brain cells occurred at 50 mg/kg BrdU and above 41 mg/kg EdU. Higher dose of 225 mg/kg BrdU or the equimolar dose of EdU did not result in any further significant increases. These results show that both markers are reliable for the detection of proliferating cells in birds, but the numbers obtained with BrdU and EdU should not be compared.

## 1. Introduction

The phenomenon of adult neurogenesis occurs in a wide variety of species including mammals and birds. The newborn cells arise from progenitor cells in specific brain regions, migrate to their ultimate destinations and differentiate. The new neurons in mammals are born in the subventricular zone (SVZ) of the lateral ventricles or in the subgranular zone of the dentate gyrus in hippocampus [1]. The proliferating cells in birds are located in the walls of the lateral ventricle in the ventricular zone (VZ) [2].

The accurate and effective labeling of the newborn cells is critical for the study of neurogenesis. Although there are several other methods for the detection of DNA synthesis [3], the marker of choice in recent years is the thymidine analogue 5-bromo-2′-deoxyruidine (BrdU) that is incorporated into the DNA during the S-phase of the cell cycle [4]. BrdU is immunohistochemically detected and allows co-localization with other cell type markers. A drawback is that it requires a DNA denaturation (usually using hydrochloric acid) in order to unmask the epitopes recognized by the primary antibody. Other methods detecting DNA synthesis involve using halogenated thymidine analogues 5-iodo-2′-deoxyuridine (IdU), 5-chloro-2′-deoxyuridine (CldU), and a relatively newer marker 5-ethynyl-2′-deoxyuridine (EdU) [5]. The cell populations labeled by IdU and CldU can be detected by their respective antibodies and require DNA denaturation [6]. The incorporated EdU, on the other hand, can be detected via a fluorescent azide that can penetrate into the double-stranded DNA, and reacts with the ethynyl group of EdU [5]. This leads to greater sensitivity, rapid and reliable detection without the adverse effects on tissue [7]. EdU has a less severe effect on the cell cycle than CldU [8], but repeated doses and/or long-term experiments can impact the biology of cells [3,9].

When labeling two populations of cells by two makers, there is a possibility of cross-reactivity. The most potent BrdU antibodies label also IdU and CldU [10]. However, the two branches of labeled cells can be distinguished in the same animal by combining specific BrdU antibodies and EdU detection [11,12,13]. Thus, combining BrdU and EdU seems to be more reliable than combining two halogenated thymidine analogues.

The saturating doses of BrdU necessary to detect the entire population of dividing cells in the adult rat dentate gyrus are 200–300 mg/kg of body weight [14,15]. In comparison, doses of 50–100 mg/kg BrdU are sufficient to label the vast majority of S-phase proliferative populations in mouse SVZ [16] and 50 mg/kg EdU result in near saturation labeling of proliferating cells in mouse dentate gyrus [17]. Both EdU and BrdU detect proliferating cells with similar sensitivity [17]. High doses and/or repeated administration, however, cause cellular toxicity [8,18]. To minimize these adverse effects, doses of 50–100 mg/kg injected once or a few times have been used in adult rodents, non-human primates, and birds [19,20]. The saturating doses of BrdU or EdU to obtain reliable quantitative data in birds are not known. Neither is it known whether BrdU and EdU are equally sensitive in birds. Therefore, we aimed to compare the effectivity of the equimolar doses of BrdU and EdU and find a magnitude of cell proliferation in the avian brain. We obtained tissue also from liver and intestine and compared the results from the brain with these organs.

## 2. Materials and Methods

### 2.1. Animals and Housing

Thirty-three male zebra finches (*Taeniopygia guttata*) 6–12 months old were used in this experiment. The birds were housed in the animal facilities of the Centre of Biosciences, Slovak Academy of Sciences, with light:dark cycle 14:10 h and food and water *ad libitum*. They were kept in the same group aviary with other males from the age of 3 months. All procedures followed the European Union ethical guidelines and were approved by the State Veterinary and Food Administration of the Slovak Republic.

### 2.2. BrdU/EdU Injections and Tissue Processing

The birds were placed into sound attenuating chambers and stayed there for one week. On the day of sacrifice, they received one injection of BrdU (Sigma Aldrich; concentration 10 mg/mL) or equimolar doses of EdU (Carl Roth; concentration 10 mg/mL) i.m. The injected doses were 10, 50, and 225 mg/kg for BrdU (*n* = 3,6,4 birds, respectively) or 8, 41, and 184 mg/kg for EdU (*n* = 3,4,4 birds, respectively). The birds were assigned to the groups so that the age was balanced and there was no age difference (ANOVA, *p* = 0.132). In other birds, 50 mg/kg BrdU and 41 mg/kg EdU were administered in the same animals. The drugs were injected at the same time (*n* = 5 birds) or with 6 h delay (BrdU and then EdU: *n* = 2 birds, EdU and then BrdU: *n* = 2 birds). The birds were sacrificed 2 h after the last injection by lethal dose of isoflurane anesthesia followed by transcardial perfusion using phosphate buffered saline (0.1 M PBS) and 4% paraformaldehyde. The brains were dissected and in birds injected by both BrdU and EdU, liver and duodenum were also obtained. The tissue was postfixed for 5 h and immersed in 20% and 30% sucrose. Then, it was frozen in Tissue-Tek OCT compound (Sakura, Japan). The brains were cut coronally using the Leica 1800 cryocut (Leica) into 30 μm free-floating sections stored in PBS with 0.1% sodium azide at 4 °C. Liver and intestine were cut into 30 μm sections, mounted on silanated slides, and stored at −20 °C.

### 2.3. Immunohistochemistry

The sections were washed 3 × 5 min in PBS. For BrdU staining, the DNA was denatured in 2 N HCl for 7 min at 37 °C, the reaction was stopped in 0.1 M borate buffer for 3 min and the sections were washed for 3 × 5 min in PBS. The non-specific binding was blocked for 1 h in blocking solution containing 0.1% bovine serum albumin and 0.3% Triton X-100 in PBS. The sections were then incubated with primary antibodies rat polyclonal anti-BrdU (OBT 0030, Accurate Chemical, Westbury, NY, USA, diluted 1:500 in blocking solution) and mouse polyclonal anti-NeuN (MAB377, Milipore, Bedford, MA, USA, diluted 1:500; neuronal marker) or with mouse monoclonal anti-BrdU (MoBU-1, Invitrogen, Carlsbad, CA, USA, diluted 1:1000; in 5 birds also 1:200), for 48 h at 4 °C. Then, the sections were washed 3 × 5 min in PBS and incubated with secondary antibodies donkey anti-rat IgG conjugated with Cy3 (Jackson ImmunoResearch, USA), donkey anti-mouse IgG conjugated with Alexa 488 or donkey anti-mouse IgG conjugated with Alexa 594 (both from Invitrogen, Carlsbad, CA, USA) for 2 h in dark. The secondary antibodies were diluted 1:500 in the blocking solution. The sections were washed 3 × 5 min in PBS, rinsed in deionized water, and coverslipped with the Vectashield mounting medium (Vector Laboratories, Burlingame, CA, USA).

For EdU staining, the same procedure was performed except the DNA denaturation steps. To identify a possible effect of the denaturation on the staining, one series of sections was stained including the HCl treatment. The donkey anti-mouse IgG conjugated with Alexa 594 (Invitrogen, Carlsbad, CA, USA) was used as a secondary antibody for immunohistochemical labeling of NeuN. After that, EdU was stained via an azide-click reaction. The commercially available kit EdU-Click 488 (Carl Roth, Karlsruhe, Germany) was used. The sections were washed 3 × 5 min in PBS, incubated in EdU labeling cocktail for 30 min in dark, washed 3 × 5 min in PBS, rinsed in deionized water and coverslipped with the Vectashield mounting medium (Vector Laboratories, Burlingame, CA, USA).

In birds with both markers that were injected, BrdU immunohistochemistry was followed by EdU staining in the same sections. We did not find any difference when we stained EdU first in adjacent sections (*n* = 2 birds). The primary mouse monoclonal anti-BrdU (MoBU-1, Invitrogen, Carlsbad, CA, USA) was used. Liver and intestine on slides were stained using the same procedure, but the sections were first fixed to slides for 1 min using 4% paraformaldehyde and the concentration of primary and secondary antibodies increased to 1:500 and 1:250, respectively.

### 2.4. Analysis and Statistics

The photomicrographs were taken using the Leica microsystems software LAS AF6000 connected to the fluorescent microscope Leica DM5500 and the Leica DFC 340 FX camera. The 10× lens was used. The BrdU^+^ and EdU^+^ cells were quantified in VZ at four anatomical levels according to the presence of other brain structures in the section—vocal nucleus Area X (about 300 μm caudally from its anterior end; VZ_X_), *tractus septopallio-mesencephalicus* (VZ_TSM_), *commisura anterior* (VZ_CA_), and vocal nucleus HVC (at its anterior level; VZ_HVC_; see [12]). The labeling of neurons by NeuN was performed to identify the VZ and the location of Area X and HVC as the neuronal morphology and density in vocal nuclei differ from the surrounding areas. The Adobe Photoshop CS6 software was used for quantification and the number of cells was recalculated per mm length. The counts were taken from at least three sections per level of VZ. Since there was no difference between the counts taken in the left and right hemispheres (Wilcoxon test; *p* = 0.73 for BrdU stained with OBT0030; *p* = 0.82 for BrdU stained with MoBU-1; *p* = 0.76 for EdU); the numbers were averaged per bird.

The statistical analysis was performed using ANOVA with the following Fisher’s LSD test. The factors were marker (BrdU, EdU), dose (10, 50, 225 mg/kg), and brain region (VZ_X_, VZ_CA_, VZ_TSM_, VZ_HVC_). Paired *t*-tests were used to compare the cell counts obtained by two anti-BrdU antibodies, BrdU and EdU as well as to compare two concentrations (1:1000 and 1:200) of the MoBU-1 antibody and EdU with and without DNA denaturation.

## 3. Results

### 3.1. Saturation Analyses

**Figure 1:**
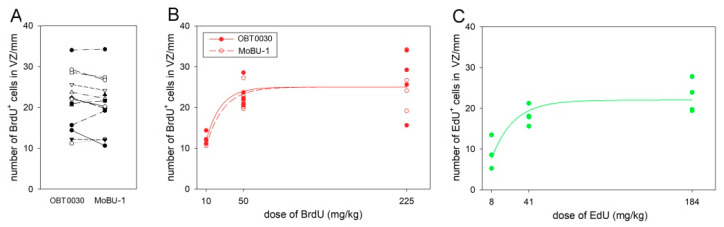
Comparison of two antibodies against BrdU (**A**) and saturation in the number of BrdU (**B**) and EdU (**C**) labeled cells in the VZ. (**A**) The number of BrdU^+^ cells was obtained using two antibodies, OBT 0030 and MoBU-1. Each line connects the values from the same animal. (**C**) The doses of EdU are equimolar to the doses of BrdU (in **B**). Each point represents an average number of labeled cells per mm length in one animal. BrdU showed saturation at higher dose than EdU.

The number of BrdU^+^ cells can depend on the antibody used for the detection, and therefore we first compared two of the most potent antibodies, OBT0030 and MoBU-1. The paired *t*-test revealed that there is no significant difference in fluorescent labeling between these two antibodies (*p* = 0.36; Figure 1A). Since the MoBU-1 antibody has been used in different concentrations in different studies [12,13], we examined whether it might affect the number of labeled cells. We found that the MoBU-1 antibody stained a similar number of cells at concentrations 1:1000 and 1:200 (*p* = 0.52, paired *t*-test).

Next, we looked at whether there is a saturation in BrdU or EdU labeling. We found that the increasing doses of BrdU and EdU led to increases in the numbers of labeled cells in the VZ. The number of BrdU^+^ cells initially increased from 10 mg/kg, and at about 50 mg/kg it reached a plateau. The saturation curves using both anti-BrdU antibodies were similar (Figure 1B). The number of EdU^+^ cells also initially increased and then reached a plateau after 41 mg/kg (Figure 1C), which is the equimolar dose to 50 mg/kg of BrdU.

### 3.2. Comparison of Doses

**Figure 2:**
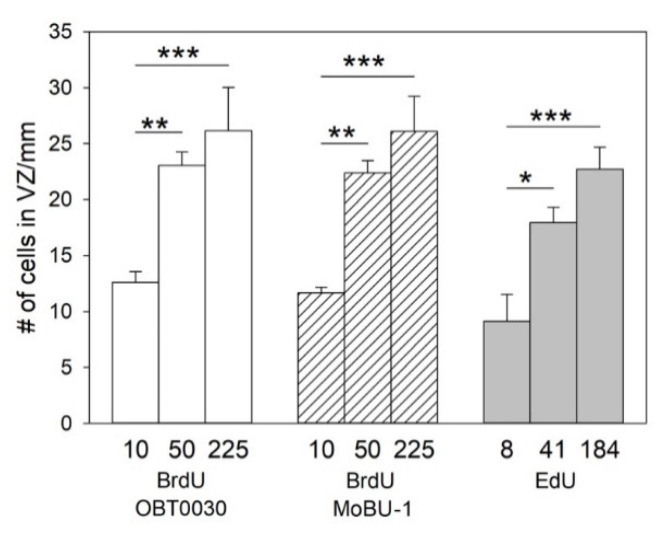
The mean number of BrdU^+^ or EdU^+^ cells in the VZ. BrdU was detected by OBT0030 and MoBU-1 antibodies. The x-axis shows doses of BrdU and EdU (equimolar to the doses of BrdU). Each bar represents a mean and SEM. The dose 50 mg/kg of BrdU resulted in more labeled cells than the dose 10 mg/kg but did not differ from the dose 225 mg/kg. The same applies for equimolar doses of EdU. * is *p* < 0.05, ** is *p* < 0.01, *** is *p* < 0.001. ANOVA and Fisher’s LSD test.

The two-factor ANOVAs showed significant effects of the proliferation marker (BrdU, EdU; *p* = 0.04 for both OBT0030 and MoBU-1 vs. EdU) as well as dose (10, 50, 225 mg/kg; *p* < 0.001 for both comparisons) on the number of labeled cells in the VZ. BrdU labeled a higher number of cells than EdU. The following Fisher’s LSD tests showed that the doses 50 and 225 mg/kg labeled significantly more cells than the dose 10 mg/kg (Figure 2). However, the dose 225 mg/kg did not label more cells than the dose 50 mg/kg. This applied for both BrdU and EdU (Figure 2).

Since the labeling of BrdU requires DNA denaturation, we stained EdU again in adjacent sections and used HCl treatment. We found no significant difference in the number of EdU^+^ cells in VZ when compared the conditions with and without the treatment with HCl (*p* = 0.457, paired *t*-test).

**Figure 3:**
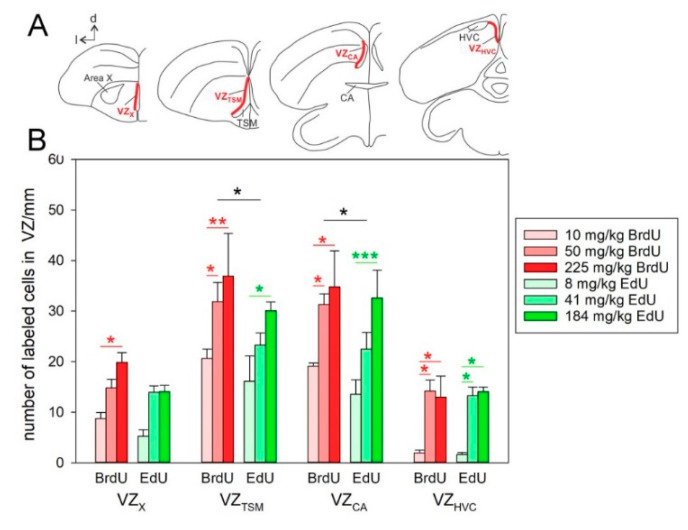
The schematic drawings of the brain (**A**) and the number of cells labeled by different doses of BrdU or EdU in different parts of VZ (**B**). (**A**) The schematic drawings show one hemisphere in coronal sections with the locations of parts of ventricular zone (VZ) that were used for quantification (highlighted red). Area X and HVC—vocal nuclei, CA—*commisura anterior*; TSM—*tractus septopallio-mesencephalicus.* (**B**) Graph showing quantification of BrdU (red bars) and EdU (green bars). Each bar represents mean and SEM. The highest numbers of labeled cells were found in VZ_TSM_ and VZ_CA_ and there was a difference between the numbers of BrdU^+^ and EdU^+^ cells in these two regions. Red stars are for the comparisons within BrdU, green stars are for the comparisons within EdU, black stars are for the comparisons between BrdU and EdU. * is *p* < 0.05, ** is *p* < 0.01, *** is *p* < 0.001. ANOVA and Fisher’s LSD test. BrdU was detected by the antibody OBT0030.

In the last phase, we performed a more detailed analysis that included the four parts of the VZ. We found significant effects of marker (BrdU, EdU; *p* < 0.01 for OBT0030 vs. EdU, *p* = 0.02 for MoBU-1 vs. EdU), brain region (*p* < 0.001 for both comparisons), and dose (*p* < 0.001 for both comparisons) on the number of labeled cells. The Fisher’s LSD test identified differences among the individual anatomical levels of VZ. The highest number of BrdU^+^ and EdU^+^ cells was detected in VZ_TSM_ and VZ_CA_ (Figure 3). The numbers in these regions were significantly higher than those in VZ_X_ and VZ_HVC_ (*p* < 0.001 for all comparisons).

**Figure 4:**
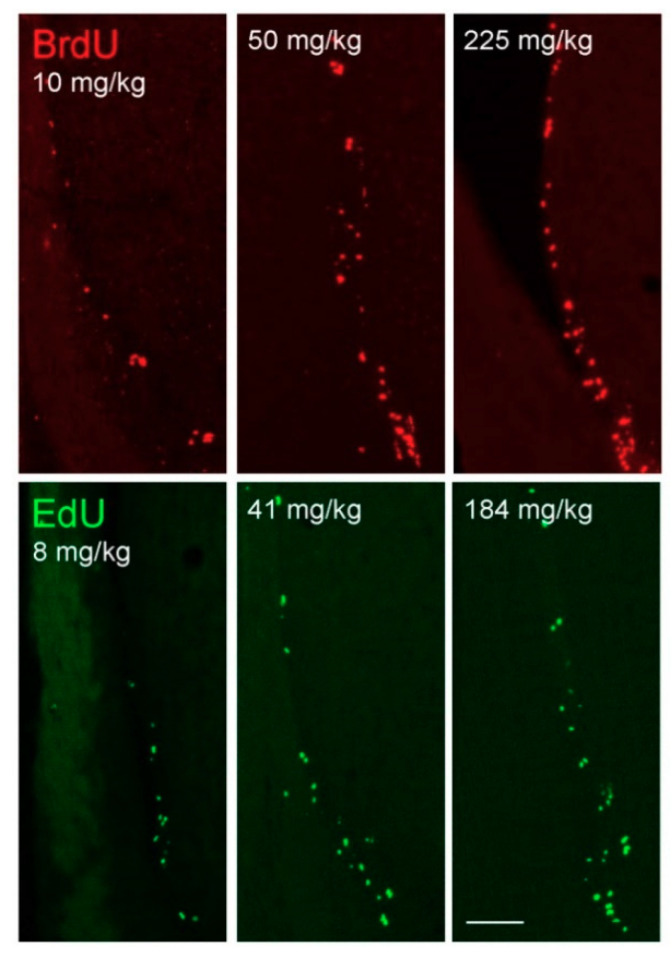
Fluorescent immunohistochemical labeling of BrdU (red) and fluorescent detection of EdU (green) after injection of different doses of the proliferation markers. Images show VZ_TSM_ after 10, 50, and 225 mg/kg BrdU or equimolar doses of EdU. BrdU was detected by the antibody OBT0030. The dose 50 mg/kg of BrdU resulted in more labeled cells than the dose 10 mg/kg but did not differ from the dose 225 mg/kg. The same applies for equimolar doses of EdU. Medial is left, lateral is right. The white scale bar in the last image represents 100 μm.

The differences among doses at different levels of VZ (Figure 3) corresponded to the previous comparison for the whole VZ (Figure 2). The higher doses resulted in more labeled cells and there were differences between the doses of 10 and 50 mg/kg or 10 and 225 mg/kg of BrdU (Figure 3, red stars; Figure 4) and the equimolar doses within EdU (Figure 3, green stars; Figure 4). However, there was no difference between the doses 50 and 225 mg/kg of BrdU or the equimolar doses of EdU. When comparing across markers, there was significantly more cells after 50 mg/kg of BrdU than after the equimolar dose of EdU in VZ_TSM_ and VZ_CA_ (Figure 3, black stars; Figure 4).

### 3.3. Comparison of BrdU and EdU Labeling in the Same Animal

**Figure 5:**
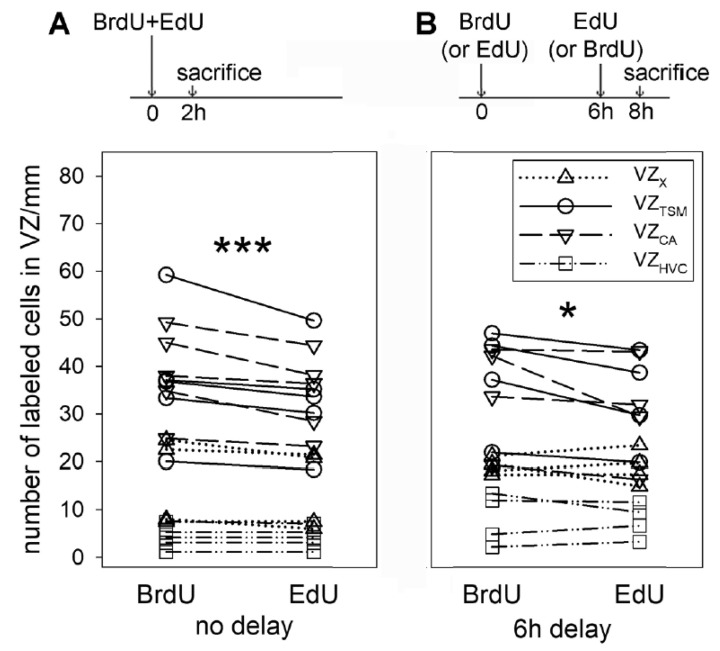
Scheme of BrdU and EdU injections and quantification of labelled cells in VZ of the same birds. Five birds in (**A**) received BrdU and EdU at the same time. Two birds in (**B**) received BrdU and 6 h later EdU, 2 other birds received EdU and 6 h later BrdU. Each line in the graphs connects the values from the same animal. The same symbol/line represents values obtained in VZ_X_, VZ_TSM_, VZ_CA_, or VZ_HVC_. BrdU labeled more cells than EdU. * represents *p* < 0.05, *** represents *p* < 0.001, paired *t*-tests.

**Figure 6:**
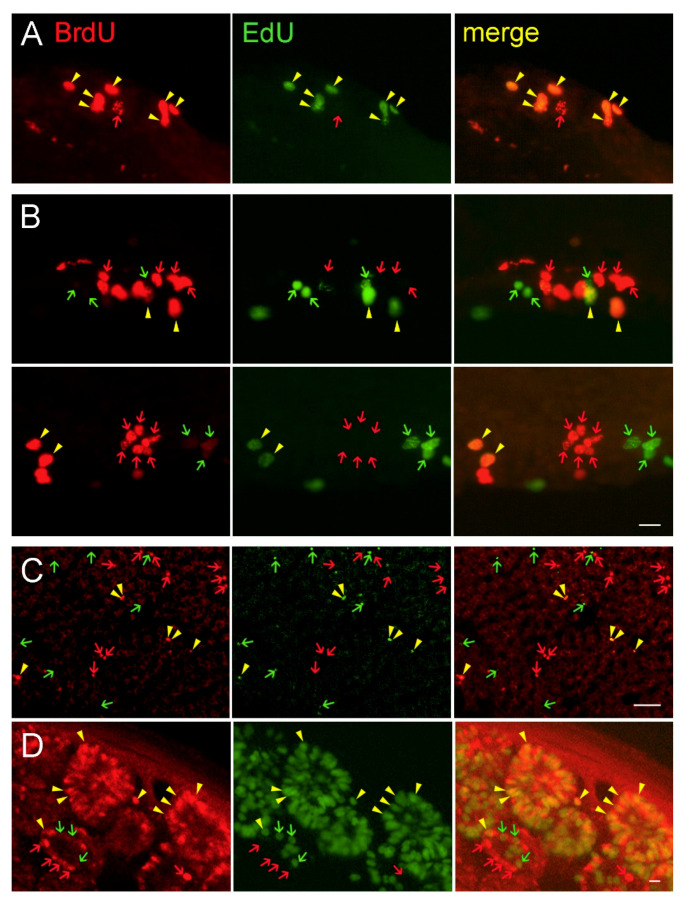
Fluorescent immunohistochemical labeling of BrdU (red) and fluorescent detection of EdU (green) in the brain (**A**,**B**), liver (**C**), and intestine (**D**) after injection of the proliferation markers in the same animal. (**A**) BrdU^+^ and EdU^+^ cells in VZ when the markers were injected simultaneously. (**B**) BrdU^+^ and EdU^+^ cells in VZ when the markers were injected with 6 h delay. Upper row—BrdU injection followed by EdU, lower row—EdU injection followed by BrdU. BrdU^+^ and EdU^+^ cells in liver (**C**) Liver and intestine (**D**) when the markers were injected with 6 h delay (BrdU first). BrdU was detected by the antibody MoBU-1. Red and green arrows point to the single labelled cells, yellow arrowheads point to the double labelled cells. The images document the results shown in Figure 5. The white scale bar represents 10 μm in **A** and **D** and 50 μm in **C**.

In order to further confirm that BrdU labels a higher amount of cells than EdU, we injected both markers simultaneously (50 mg/kg of BrdU and the equimolar dose 41 mg/kg of EdU in five birds; Figure 5A, scheme). We found that the majority (89.4 ± 2.7%) of the labeled cells in VZ showed co-localization of BrdU and EdU. Some cells, however, were labeled only by BrdU or only by EdU (9.3 ± 2.2% and 1.3 ± 0.8%, respectively; Figure 6A). Counting BrdU^+^ and EdU^+^ cells in the individual parts of VZ revealed a higher number of BrdU^+^ cells in comparison to EdU^+^ ones (Figure 5A; *p* < 0.001, paired *t*-test).

To unequivocally exclude the possibility of staining overlap, we injected BrdU in another group of birds and then EdU with a 6 h delay (Figure 5B, scheme; the order of markers was alternated in different birds). We found separate populations of BrdU- and EdU-labeled cells but there were also cells with co-localized markers (6.9 ± 0.7%; Figure 6B). Significantly more cells were labeled by BrdU than by EdU (Figure 5B; *p* < 0.05, paired *t*-test).

Similar pattern of staining was found also in other organs with high cell proliferation, liver and duodenum (Figure 6C,D). We found a significantly higher number of BrdU^+^ and EdU^+^ cells in liver when the proliferation markers were injected simultaneously (*p* < 0.05; paired *t*-test), but not when they were injected with a delay (*p* = 0.443, paired *t*-test). The percentage of co-localized cells was 45.5 ± 6.6 when BrdU and EdU were injected simultaneously and 9.3 ± 2.3 when they were injected with a 6 h delay (Figure 6C). When looking at the intestine, proliferating cells were found in duodenal crypts. BrdU and EdU were found co-localized in the cells in both groups of birds, even when the second marker was injected 6 h after the first one (Figure 6D). We were not able to quantify the exact numbers of labelled cells due to high background.

## 4. Discussion

Although there are several methods available for the detection of DNA synthesis and cell proliferation, the protocols based on BrdU and EdU are relatively simple, cheap, and represent an optimal choice for most studies [3]. These markers have been used to label neuronal populations arising at different times in the same individuals, but comparison of their effectivity in birds absents. Here, we used different doses of BrdU and EdU and examined the numbers of labeled cells in the neurogenic VZ and liver.

In the first phase, we aimed to identify a dose that is able to detect the highest number of cells in VZ. We found that the number of cells increased with increasing doses and it reached plateau at the dose 50 mg/kg BrdU and above 41 mg/kg EdU (equimolar dose to 50 mg/kg BrdU). According to Cameron and Mckay (2001), doses 100, 50, and 25 mg/kg BrdU label 60%, 45%, and 5% of all the proliferating cells labeled by 300 mg/kg BrdU in rat dentate gyrus. On the other hand, in adult mice, only 50–100 mg/kg BrdU leads to the labeling of all proliferating cells in subventricular zone [16] and 150 mg/kg BrdU is a saturating dose for labeling all newborn cells in the dentate gyrus of hippocampus [21]. Similarly, Zeng et al. (2010) detected the highest number of cells using 50 mg/kg EdU in the mouse dentate gyrus. Thus, our results in zebra finch correspond more with the doses of BrdU and EdU necessary to label the highest number of cells published in mouse rather than in rat.

BrdU is transported to the brain similarly as thymidine via nucleoside transport systems in the blood–brain barrier [22] and these transporters have low affinity and high capacity [23]. We assume that EdU uses the same mechanism to enter the brain. It is possible that the plateau in the number of labeled cells occurs due to saturation of the nucleoside transporters [15]. The permeability of the blood–brain barrier is particularly tight in rats [24] and density, affinity and conformations of the transport system vary among species [25]. Therefore, the doses necessary for saturation in the number of labeled cells can differ among species.

The detectability of the proliferating cells depends also on the fluorescent intensity. Lower doses of the markers result in lower fluorescent intensity and therefore it was suggested that saturating doses should be used to obtain reliable quantitative data [17]. Although the highest numbers of cells in our study were found after 225 mg/kg BrdU and 184 mg/kg EdU, these numbers were not significantly different from those obtained after 50 mg/kg BrdU and 41 mg/kg EdU, respectively. Similarly, the saturation curve shows that 50 mg/kg BrdU leads to saturation in the number of labeled cells. In case of EdU, the saturation occurs at slightly higher doses, 41–80 mg/kg.

Some of the disadvantages of BrdU are that it labels not only the proliferating cells but also cells during their repair, and a high dose causes apoptosis [8,18]. We suppose that the toxicity of the high doses used in our study did not affect the results, as the markers label cell in the S-phase and the cell cycle was not completed yet. The S-phase of the cell cycle in the SGZ of mice takes approximately 7.6 h [26]. The percentage 6.9% of the double labeled BrdU^+^/EdU^+^ cells when the second marker was injected 6 h later suggests that the S-phase duration in birds is similar to that in mice [26]. Although Cameron and McKay [15] did not show BrdU toxicity in adult rodents, other studies verified that a dose of BrdU higher than 60 mg/kg in rats [19] can cause cytotoxic changes in the animal. It is possible, though, that BrdU is less toxic in adults than in embryos or juveniles [27]. Generally, doses 50–100 mg/kg of BrdU or EdU are used for repeated injections.

Although EdU is faster and simpler to use, we found that it is not equivalent to BrdU in birds. There were more cells labeled by BrdU than by EdU and the difference was significant at 50 mg/kg BrdU and the equimolar dose of EdU in VZ_TSM_ and VZ_CA_ with the highest numbers of proliferating cells. We found the higher number of BrdU^+^ cells also when the proliferation markers were injected in the same birds, either together or with delay. In comparison, equimolar doses of thymidine analogues did not label the same number of cells in VZ in other studies either [10,12]. Zeng et al. [17] report comparable labeling of EdU and BrdU, but they only used a dose of 50 mg/kg EdU and the equimolar dose of BrdU in the same animal. In that study, Zeng et al. stained BrdU and EdU in the same sections, thus the DNA was denatured even for EdU staining. Here, we also denatured DNA before EdU staining, in birds treated only with EdU as well as in birds with both markers injected. We found no significant effect of this treatment on the number of EdU^+^ cells. Therefore, we believe that the denaturation steps do not affect the results.

Differences in the number of labeled cells after equimolar doses of BrdU and EdU in our study can be caused by different detections of these thymidine analogues. It is possible that there are different rates for the antibody against BrdU and the fluorescent azide for EdU to enter a cell. Moreover, there are substantial differences among BrdU antibodies in their potency to label cells. We used the antibodies OBT0030 from Accurate and MoBU-1 from Invitrogen that are the most sensitive to BrdU detection in cells but were not compared [10,11]. Our results show that the cell counts obtained with these antibodies are comparable. According to our previous results, when both markers are applied in the same bird, the concentration of MoBU-1 1:200 leads to cross-reactivity and labels also EdU^+^ cells [12]. Here, we show that the 1:1000 dilution of the antibody reduces this problem while the 1:200 and the 1:1000 dilutions labeled a similar number of cells.

The differences in BrdU uptake can also be caused by different clearances of BrdU and EdU. Most of the studies utilizing BrdU presume that this marker is available for 2 h after the injection. This is based on the information obtained mostly for the older marker, tritiated thymidine [28,29,30] and tritiated BrdU [30]. The availability of the markers increases with the increasing dose. A newer in vivo study reports, however, that BrdU is no longer available for incorporation into DNA 30–60 min after injection of 100 mg/kg in songbirds, quails and mice [31]. The clearance rate for EdU is not known. If the higher number of labeled cells after BrdU than EdU was explained by their clearance rates, then EdU would have to be available for shorter time than 30 min.

Our experiments further showed that the highest cell proliferation in the neurogenic zone of zebra finch occurred in the central regions of VZ, VZ_TSM_ and VZ_CA_, and that the lowest cell proliferation occurred in the rostral and caudal parts, VZ_X_ and VZ_HVC_. These results correspond with the highest number of proliferating cells found in the central region of VZ in the caudomedial striatum in canaries [32,33].

Last but not least, we found more BrdU than EdU labelled cells in liver 2 h after the injection of equimolar doses of both proliferation markers. This suggests that, similar to the brain, BrdU is also labeling more effectively than EdU in the liver. The percentage of the double-labeled cells when BrdU and EdU were injected simultaneously was considerably lower than that in the brain. The percentage 9.3% of co-localized cells in the group where the second marker was injected 6 h after the first one is consistent with the known 9 h duration of DNA synthesis in the rat liver cells [34]. The location of the proliferation markers in the duodenal crypts in our study is consistent with the location in mice 2 h after BrdU injection [35].

## 5. Conclusions

Our study shows that both BrdU and EdU are reliable markers of cell proliferation in VZ of zebra finches. Although the staining protocol for EdU is faster and simpler, it detects fewer cells than the equimolar doses of BrdU. The dose detecting the highest number of cells in songbirds is lower than that in rats and comparable to that in mice. Two generations of cells can be detected when BrdU and EdU are injected at different time-points in the same animal, but the numbers of BrdU and EdU labeled cells should not be compared. The number of labeled cells can only be compared between different experimental treatment groups, within the same proliferation marker.
